# A Physiotherapy Treatment Plan for Post-COVID-19 Patients That Improves the FEV1, FVC, and 6-Min Walk Values, and Reduces the Sequelae in 12 Sessions

**DOI:** 10.3389/fresc.2022.907603

**Published:** 2022-05-30

**Authors:** Silvia Denise Ponce-Campos, Juan Manuel Díaz, Daniela Moreno-Agundis, Ana Laura González-Delgado, Paulina Andrade-Lozano, Francisco Javier Avelar-González, Eduardo Hernández-Cuellar, Fernando Torres-Flores

**Affiliations:** ^1^Unidad Medico Didáctica, Universidad Autónoma de Aguascalientes, Aguascalientes, Mexico; ^2^Unidad de Medicina Interna, Adscripción al Servicio de Neumología, Hospital General ISSSTE Aguascalientes, Aguascalientes, Mexico; ^3^Departamento de Medicina, Universidad Autónoma de Aguascalientes, Aguascalientes, Mexico; ^4^Departamento de Fisiología y Farmacología, Universidad Autónoma de Aguascalientes (UAA), Aguascalientes, Mexico; ^5^Departamento de Morfología, Universidad Autónoma de Aguascalientes (UAA), Aguascalientes, Mexico

**Keywords:** post-COVID-19, respiratory function, physiotherapy, 6-min walk test, SARS-CoV-2, spirometry

## Abstract

Severe acute respiratory syndrome coronavirus 2 (SARS-CoV-2) is the causal agent of Coronavirus disease 2019 (COVID-19), a pandemic disease declared in 2020. The clinical manifestations of this pathology are heterogeneous including fever, cough, dyspnea, anosmia, headache, fatigue, taste dysfunction, among others. Survivors of COVID-19 have demonstrated several persistent symptoms derived from its multisystemic physiopathology. These symptoms can be fatigue, dyspnea, chest pain, dry and productive cough, respiratory insufficiency, and psychoemotional disturbance. To reduce and recover from the post-COVID-19 sequelae is fundamental an early and multifactorial medical treatment. Integral post-COVID-19 physiotherapy is a tool to reduce dyspnea, improve lung capacity, decrease psychoemotional alterations, as well as increase the muscle strength affected by this disease. Thus, the aim of this study was to establish a novel physiotherapeutic plan for post-COVID-19 patients, evaluating the effect of this treatment in the reduction of the sequelae in terms of lung capacity, cardio-respiratory, and muscular strength improvements. This was a cross-sectional study in which a protocol of 12 sessions in 4 weeks of physiotherapy was implemented in the patients enrolled. We conducted a medical assessment, an interview, a DASS-21 test, a spirometry, a 6-min walk test, and a hand dynamometer test to evaluate the post-COVID condition of patients before and after the sessions. A total of 42 patients participated in the program. Results of this work showed a decrease of around 50% of post-COVID-19 sequelae and an improvement in the psychoemotional status of patients. Also, we observed an increase of 7.16% in the FEV1 value and 7.56% for FVC. In addition, the maximal functional capacity increased by 0.577 METs, the 6-min walk test performance increased by 13%, and the SpO2 improved by 1.40%. Finally, the handgrip strength test showed an improvement in the left hand and right hand of 2.90 and 2.24 Kg, respectively. We developed this study to propose a novel methodology to provide information for a better treatment and management of post-COVID-19 patients.

## Introduction

Severe acute respiratory syndrome coronavirus 2 (SARS-CoV-2) is the causal agent of Coronavirus disease 2019 (COVID-19), a pathology first identified in 2019 in Wuhan, China that has spread rapidly all over the world. This virus is mainly transmitted via droplets and aerosol. Clinical manifestations are heterogeneous, in most patients including fever, cough, dyspnea, nausea, vomiting, anosmia, headache, abdominal and muscle pain, fatigue, and taste dysfunction, among others. In some cases, the most complex form leading to death is characterized by acute respiratory distress syndrome (ARDS), pneumonia, acute kidney injury, heart failure, and secondary infections (Sun et al., 2020). These signs and symptoms are derived from the physiopathology of the SARS-CoV-2 infection as a result of cell damage and/or cell death, loss of alveolar wall integrity, detachment of lung tissue, fibroblast proliferation, extensive fibrosis, hemorrhage, and thrombus (1). Clinically, the severity of the disease is classified as (1) asymptomatic (any symptoms, within the first 20 days of infection), (2) Mild (minor symptoms that do not affect daily activities), (3) moderate (some limitations in daily activities), (4) Severe (SpO2 <90% on room air, respiratory rate >30 per minute, signs of respiratory failures, use of accessory respiratory muscles, and SpO2 >90% with the support of <10 L/min of supplemental oxygen), (5) critical (SpO2 <80% on room air, SpO2 <90% with the support of >10 L/min of supplemental oxygen, mechanical ventilation, vasopressor, dialysis, and emergency bronchoscopy) (2).

The behavior of this disease in time could be classified as “acute COVID-19” or “long COVID-19.” The acute COVID-19 phase typically endures up to 4 weeks from the onset of symptoms. If the symptomatology persists for more than 4 weeks this phase is named “long COVID” (3). Survivors of COVID-19 have demonstrated several persistent symptoms derived from cellular damage, immune response, inflammatory response, and circulatory dysregulation; these symptoms can be fatigue, dyspnea, chest pain, dry and productive cough, digestive and neuropsychiatric symptoms, respiratory insufficiency, cognitive and psychoemotional disturbance. Finally, as a result of these sequelae, the patients suffer a decline in their quality of life (3, 4).

To reduce the post-COVID-19 sequelae is fundamental to an early and multifactorial treatment managed by a multidisciplinary medical staff (5). Integral post-COVID-19 physical therapy is the main tool to reduce dyspnea, improve lung capacity, decrease the level of anxiety, stress, and depression, as well as increase the muscle strength affected by this disease (6). These results are obtained due to the multiple exercise program oriented toward removing secretions in the airways and obtaining a reeducation of the respiratory pattern (control of breathing, chest expansion, and expiration) in order to improve the respiratory volume and the oxygen saturation. The Debit Controlled Inspiratory Exercises (EDIC) and autogenic drainage are addressed to improve the ventilation-perfusion ratio (V/Q) and decreasing the fatigue, with a reduction of the tendency to develop bronchospasm (7–9). Aerobic and strengthening exercises play a key role in enhancing cardiorespiratory and musculoskeletal performance (10). These focused on restoring and improving the quality of life of the population affected by SARS-CoV-2.

Even though there is a high index of patients with this condition worldwide, the data available about the clinical benefits and protocols established in the physiotherapeutic field focused on the post-COVID-19 syndrome are not enough. Thus, the aim of this study was to establish a novel physiotherapeutic plan to determine the effects on post-COVID-19 patients, evaluating their psychoemotional condition, lung capacity, cardiorespiratory condition, and muscle strength. We seek to propose this methodology based on physiotherapy to improve the quality of life of the COVID-19 survivors.

## Materials and Methods

This was a cross-sectional study conducted by the Medical Didactic Unit at the Universidad Autónoma de Aguascalientes in the Physical Therapy Unit of this university, from April to September 2021. This study was approved by the University ethics committee (CIB-UAA-47). An informed consent was shown and accepted by all the patients participating in the study. The patients included in this study were diagnosed with SARS-CoV-2 infection by the antigen test at least 1 month before starting the protocol. The population selected ranged in age from 18 to 99 years without gender selection and with clinical stability to perform active mobilization. Patients with stroke, neurodegenerative diseases, and additional uncontrolled cardio-respiratory diseases were excluded (5, 11–13).

**Figure 1:**
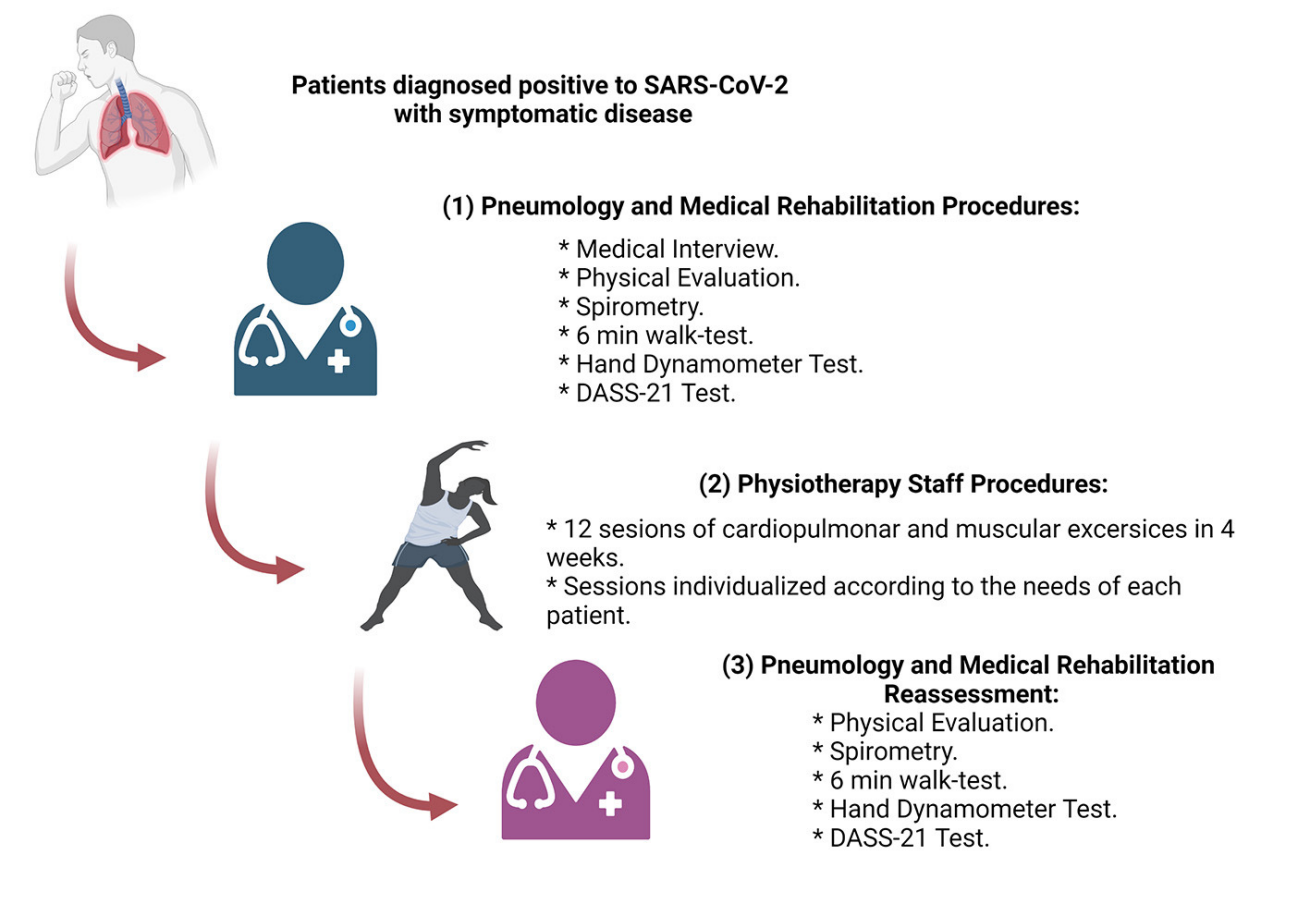
Schematization of the methodology and the sequence followed in this study.

This study was divided into three steps (Figure 1): (1) Patients enrolled, were interviewed, and physically evaluated (physical examination) by the Pneumology and Physiatry area. To measure the psycho-emotional state of the patients a Depression, Anxiety, and Stress Scale - 21 Items (DASS-21) test self-report questionnaire was performed, and the patients were classified according to the scores obtained. Briefly, The Depression scale score was divided into mild (5–6 scores), moderate (7–10 scores), severe (11–13 scores), and extremely severe depression (14–21 scores). The Anxiety scale was mild (2–4 scores), moderate (5–7 scores), severe (8–12 scores), and extremely severe (12–21 scores). Finally, the Stress subscale into mild (8–9 scores), moderate (10–12 scores), severe (13–16 scores), and extremely severe (17–21 scores) (14–16). The physical evaluation was oriented to evaluate the physical state, persistent signs, and symptoms originating from the COVID-19 (17, 18). The respiratory function of each patient was evaluated by spirometry (NDD, Easy on-PC, #2700-3) to obtain the Forced Vital Capacity (FVC) and Forced Expiratory Volume in 1 second (FEV1) in order to calculate the FEV1/FVC ratio (19). Then, to evaluate comprehensively the cardiovascular, metabolic, musculoskeletal, and respiratory capacity in the post-COVID-19 patients, a 6-min walk test (6 MWT) was performed (20). Briefly, before performing the test, weight, blood pressure, basal heart rate, and basal oxygen saturation by pulse oximetry (Companion, #D.400.001) were obtained. The walk test was carried out in an indoor hall of 30 meters with a flat surface, walking uninterruptedly the circuit until reached the 6 min of the test. Finally, the distance was recorded, and immediately, blood pressure, heart rate, and oxygen saturation were measured with the patient seated at rest. The values were taken as previously described with modifications from the 6 MWT (21, 22) such as the Maximal Functional Capacity and the 6-min walk test performance (23–26). Finally, a hand-grip dynamometry test was carried out to observe the upper limb strength of the participants (27). Briefly, a manual dynamometer (GRIP X, #EH 101) was used to assess the grip strength, the test was conducted with the participant seated on a chair (with a back and no armrests) with the lower limbs resting on the ground. The shoulder of the limb to be tested remained adducted and neutral for rotation with the elbow flexed at 90°, the forearm neutral for pronosupination, and the wrist extension between 0 and 30° with 0–15° of ulnar deviation. During the test, constant verbal encouragement was given to the participants to use their maximum strength. The test was repeated twice for each arm and the highest value on each side was taken.

(2) A total of 12 sessions in a month were recommended. The physiotherapy sessions were conducted three times per week until the end of the program. The physiotherapeutic activities and exercises performed per week by the patients are shown in Table 1. The percentage of effort in the activities was personalized and proportional to the muscular and cardio-respiratory capacity of each patient (28, 29). All the activities were performed indoors, and the aerobic exercises were conducted using a treadmill (Sole, #S77), a stationary bike (Pro-form-450 UR), or an elliptical bike (Body Rider, #BR1830), depending on the physical capacity of each patient. (3) After completing the sessions, a medical evaluation, the spirometry, the 6-min walk test, and the hand dynamometer test was reassessed to evaluate the effects of the physical therapy on the cardiopulmonary condition and muscular strength (26, 30, 31).

**Table 1:** Physiotherapy treatment plan for post-COVID-19 patients.

**Week**	**Number of Sessions per Week**	**Physiotherapeutic Activities and Exercises**
**1**	**3**	*Breathing Exercises: Purse-lips Breathing. Diaphragmatic Breathing. Thoracic Expansion. Debit Controlled Inspiratory Exercises (EDIC). Manual Mucociliary Clearance Techniques (in case of secretions). *Active or Active-assisted Mobilizations. *Jacobson's Autogenic Relaxation Exercises. *Progressive Strengthening Exercises. *Teaching of Energy Saving Techniques.
**2**	**3**	*Breathing Exercises: Purse-lips Breathing. Diaphragmatic Breathing With Manual Resistance. Thoracic Expansion. EDIC. Sum Breathing Along With Active Mobilizations. *Aerobic Exercise Between 55–60% of Maximum Heart Rate (Treadmill, Stationary Bike, or Elliptical Bike). *Progressive Strengthening Exercises. *Balance Exercises.
**3**	**3**	*Breathing Exercises. *Aerobic Exercise at 60–65% of Maximum Heart Rate (Treadmill, Stationary Bike, or Elliptical Bike). *Progressive Strengthening Exercises. *Balance Exercises.
**4**	**3**	*Breathing Exercises. *Aerobic Exercise Between 70–75% of Maximum Heart Rate (Treadmill, Stationary Bike, or Elliptical Bike). *Progressive Strengthening Exercises *Balance and Coordination Exercises.

### Statistical Analysis

Data obtained in this study were analyzed, and the results were plotted using GraphPad Prism 8.0 software. Demographic, sequela, and DASS-21 data obtained from the patients were analyzed using descriptive statistics, evaluating the values obtained at the beginning of the program and again at the end of the physiotherapeutic sessions. The clinical data from spirometry, 6-min walk test, and hand dynamometer test were examined using descriptive statistics and the Paired *T*-test in addition to observing the behavior and differences of the data. The Difference Between Population Data Pairs (μd) was obtained and plotted (32).

## Results

### Demographic and Clinical Characteristics of the Patients

During this study, a total of 42 patients were integrated into it. The demographic characteristics of all the participants enrolled in the study are shown in Table 2. The percentage of the male patients was 55.6 and 44.4% corresponded to the female gender. The mean age was 53.5 years (Minimum 18 years, Maximum 81 years). The survey for patients with comorbidities showed that around 50.0% were smokers, 42.8% had heart disease, 26.1% had type 2 diabetes mellitus, and 19.0% had obesity. Furthermore, data focused on observing the clinical course of the COVID-19 disease showed that 69.0% of the participants had developed severe pneumonia, 4.7% had pneumonia, 16.6% had a mild illness, and only 9.5% had a critical illness. Most of the population presented the disease as an outpatient (73.8%), nine were hospitalized (21.4%), and finally, two (4.7%) patients were admitted to the Intensive Care Unit and subjected to Invasive Mechanical Ventilation for 23 and 40 days, respectively.

**Table 2:** Demographic and clinical characteristics of the patients enrolled in this study.

	**Female** **(*n* = 17)**	**Male** **(*n* = 25)**	**Total,** ***n* = 42**
Patient percentage	44.4%	55.6%	100%
Average age	52.5	54.2	$\bar{\text{x}}$: 53.35
**COMORBIDITIES DISTRIBUTION**			
Hearth disease	6	12	**18 (42.8%)**
Type 2 diabetes mellitus	2	9	**11 (26.1%)**
Obesity	3	5	**8 (19.0%)**
Asthma	1	2	**3 (7.1%)**
Chronic inflammatory lung disease	0	1	**1 (2.3%)**
Dyslipidemia	2	1	**3 (7.1%)**
Cancer	0	1	**1 (2.3%)**
Tabaquism	6	15	**21 (50.0%)**
**CLINICAL COURSE OF SARS-COV-2 INFECTION**			
Mild illness	3	4	**7 (16.6%)**
Pneumonia	0	2	**2 (4.7%)**
Severe pneumonia	11	18	**29 (69.0%)**
Critical illness	2	2	**4 (9.5%)**
Ambulatory patients	15	16	**31 (73.8%)**
Hospitalization patients	2	7	**9 (21.4%)**
Intensive care unit patients	0	2	**2 (4.7%)**
Invasive mechanical ventilation	0	2	**2 (4.7%)**
Steroid therapy	12	22	**34 (80.9%)**
Anticoagulant therapy	6	10	**16 (38.0%)**

### Assessment of the Post-COVID-19 Sequelae

**Figure 2:**
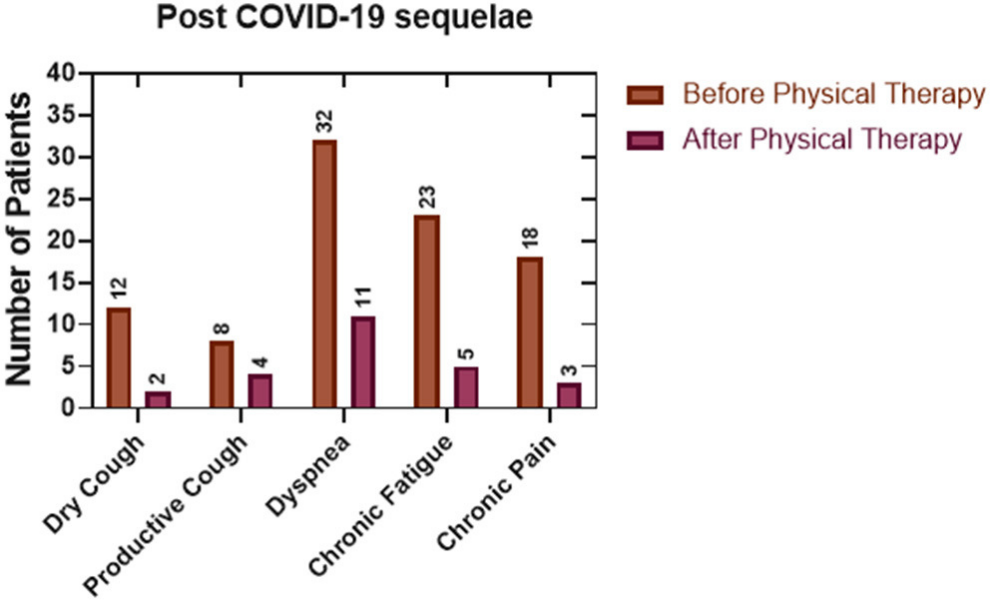
Evaluation of the post-COVID-19 sequelae before and after our physiotherapeutic plan. As a result of a comparison between the physical evaluation before and at the end of the treatment plan, it was possible to observe a reduction of the sequelae in at least half of the patients (Descriptive Statistics).

**Figure 3:**
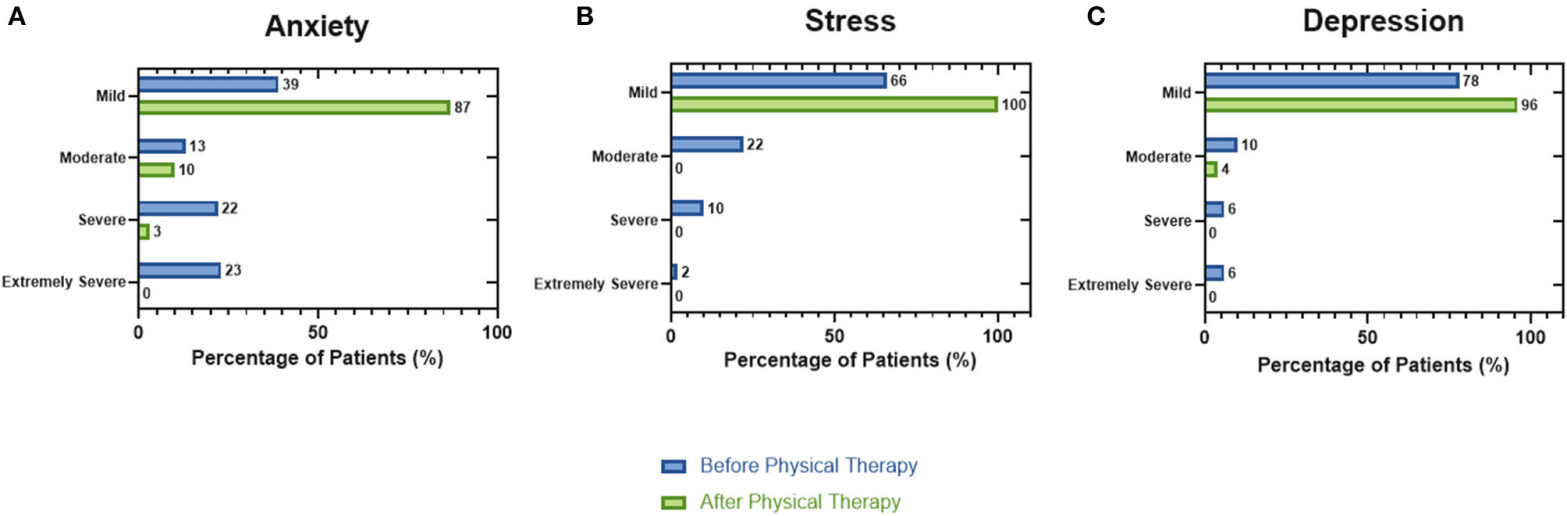
Psycho-emotional evaluation by the DASS-21 test in patients post-COVID-19 before and after the physiotherapeutic plan. **(A)** The mild anxiety levels increased 48% after completing the treatment this effect derived from the reduction of severe and moderate states. **(B)** After completing the session, all the patients showed an improvement in their stress levels. **(C)** Finally, the mild depression value was enhanced by 18% in comparison with the value obtained at the start of treatment (Descriptive Statistics).

Derived from the analysis of the data obtained from the medical interview and physical evaluation before and after the twelve sessions of physiotherapy it was possible to observe a decrease in the post-COVID-19 sequelae of the patients (Figure 2). Dyspnea was the most prevalent symptom (32 patients), and after physical rehabilitation, it was possible to reduce it to 65.6%. Also, chronic fatigue was reduced to 78.2% after completing the treatment, and chronic pain was reduced to 83.3%. Dry cough and productive cough had an 83.3 and 50.0% of reduction in the patients with these symptoms, respectively. DASS-21 test results (Figure 3) showed a reduction in the percentage of patients with moderate and severe anxiety (Figure 3A). Patients with moderate, severe, and extremely severe stress (Figure 3B) were not detected after therapy. Finally, mild depression levels were improved by 18% and moderate levels by 6% (Figure 3C).

### Spirometry Evaluation

**Figure 4:**
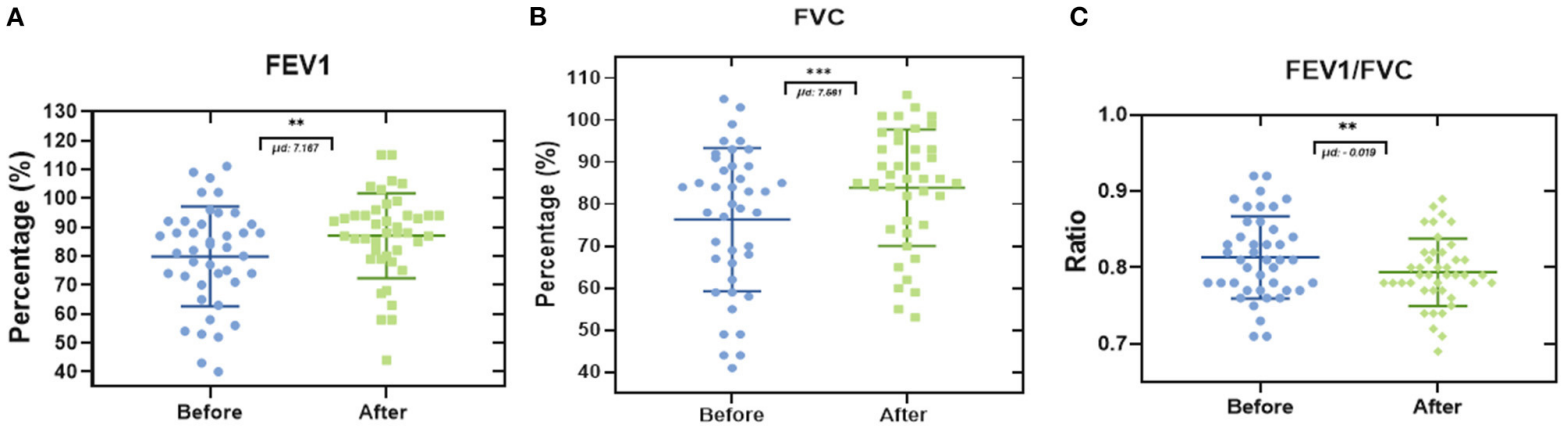
Comparison of lung capacity by spirometry in patients post-COVID-19. Each data point represents an individual patient. **(A)** Evaluation of FEV1 in the patients enrolled in this study showed an increase of 7.167% after completing the physiotherapeutic plan. **(B)** In addition, the comparison of FVC results indicates an increase of 7.561% in this value. **(C)** FEV1/FVC ratio resulted in a negative value of −0.019 (Paired *t*-test. **p ≤ 0.01, ***p ≤ 0.001. μd: difference between population data pairs).

Spirometry is the most common test used to evaluate lung function; this technique was used to observe the effect of the post-COVID-19 physiotherapy on lung capacity. Forced expiratory volume in the first second (FEV1) (Figure 4A) before therapy showed a mean of 78.86% (SD 17.29), after carrying out the treatment the mean increased to 87.02% (SD 14.71), resulting in a significant difference of 7.167%. In the case of the Forced Vital Capacity (FVC) (Figure 4B), the mean at admission was 76.32% (SD 17.01), and after the sessions was 83.88% (SD 13.87), showing a difference of 7.561%. Then, the evaluation of the FEV1/FVC ratio (Figure 4C) in this study showed a difference in the mean of −0.019.

### Six-Minute Walk Test and Hand Grip Strength

**Figure 5:**
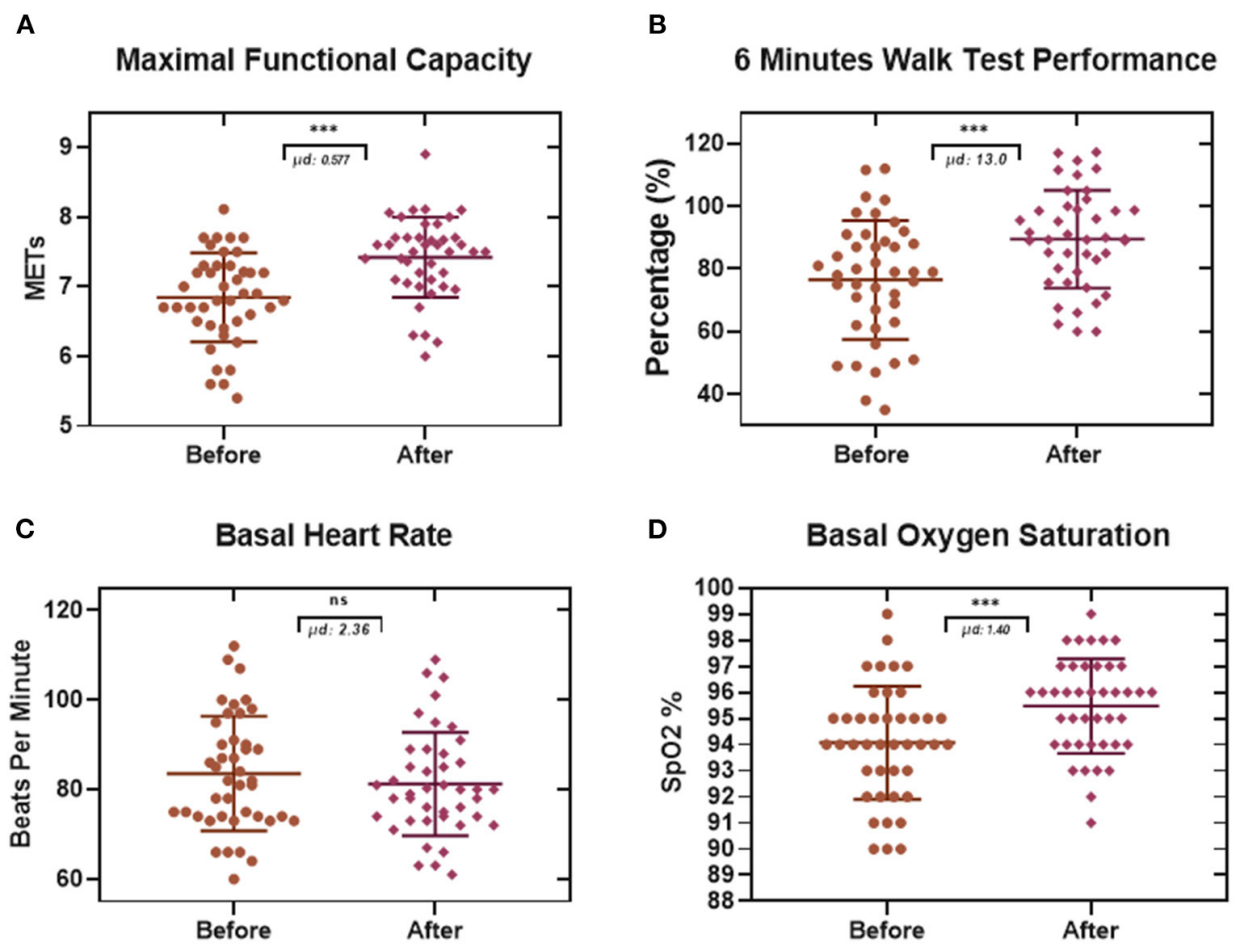
Effects of the physiotherapeutic treatment in patients post-COVID-19 evaluated by a 6-min walk test. Each data point represents an individual patient. **(A)** After completing the treatment, the Maximal Functional Capacity value increased significantly to 0.577 METs. **(B)** the percentage of the 6-min walk performance improved by 13%. **(C)** The basal heart rate presented a rise of 2.3 beats per minute, this result was not statistically significant. **(D)** Finally, the Resting Oxygen Saturation resulted in a significant increase of 1.40% (Paired *t*-test. ***p ≤ 0.001. μd: difference between population data pairs. ns: not significant).

**Figure 6:**
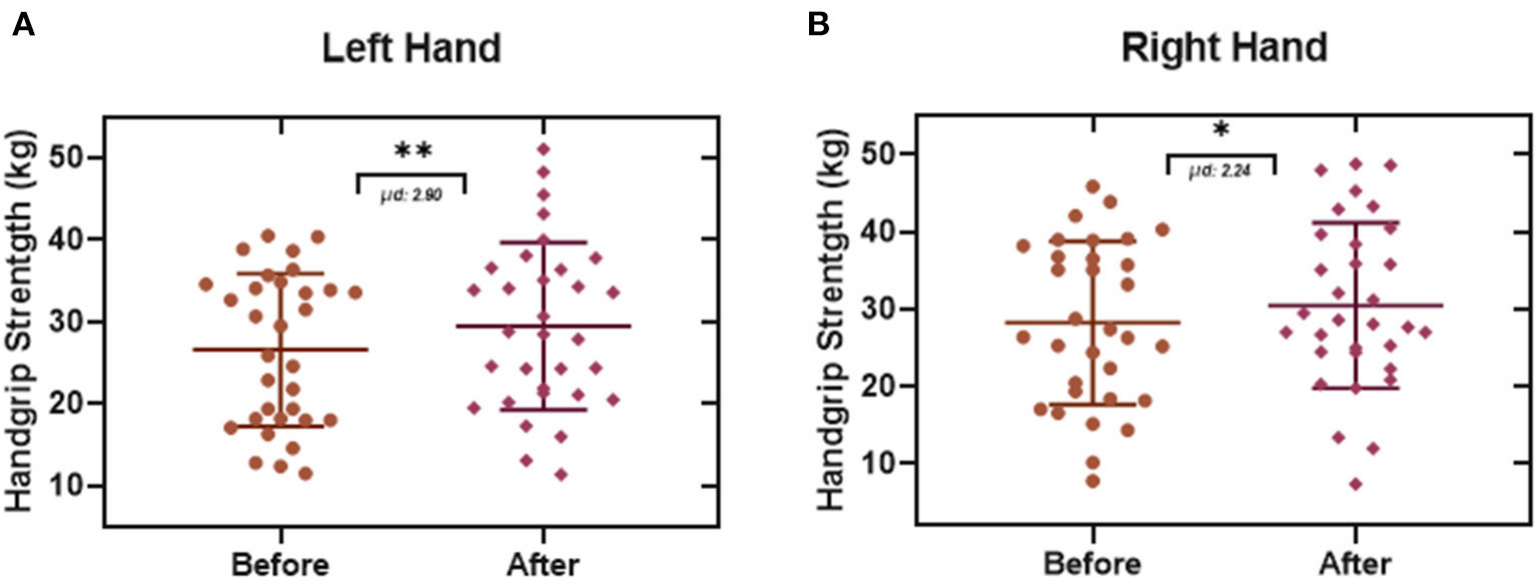
Handgrip Strength Test. Each data point represents an individual patient. Data obtained from this test showed a significant strength improvement in the upper limbs with an enhancement of 2.90 Kg in the left hand **(A)**, and **(B)** an increase of 2.24 Kg in the right hand (Paired *t*-test. *p ≤ 0.05, **p ≤ 0.01. μd: difference between population data pairs).

The comparison of the maximal functional capacity (Figure 5A) of each patient at the beginning and the end of the treatment showed a significant difference of 0.577 METs. Also, the analysis of the results of this test (Figure 5B) showed an improvement of 13% in the performance of this proof after finishing all the rehabilitation sessions. Furthermore, the evaluation of the basal heart rate (Figure 5C) in this study did not show any significant differences in our population (2.36 beats per minute). Finally, results from the basal oxygen saturation (Figure 5D) exhibited a difference of 1.40% in the SpO2 derived from a comparison between the mean oxygen saturation before (94.07 SpO2 %) and after (95.47 SpO2 %) the treatment. The handgrip strength test showed a significant improvement in both upper limbs, for the left hand (Figure 6A), it was possible to observe a difference of 2.90 Kg, and, in the case of the right hand (Figure 6B), the difference was 2.24 Kg.

## Discussion

Physical therapy is an important area for the treatment of several diseases, the intervention of this specialty is highly relevant in acute and chronic pathologies in which the main point is restoring and improving the quality of life. In post-COVID-19 patients, these benefits are avoiding or removing secretions in the airways, improving the cardio-respiratory capacity, increasing the oxygen saturation, and enhancing the muscle strengthening (13, 33).

Since the beginning of the worldwide pandemic generated by the novel coronavirus SARS-CoV-2, there has been a global effort to describe the nature of the causal agent, the physiopathology of this new disease, the appropriate treatment, and a race against time for the development of several vaccines to prevent the disease. However, the data available regarding the clinical approach of the post-COVID-19 patients are less in comparison with all the above-mentioned approaches. Ongoing Symptomatic COVID-19 (OSC) is defined as the persistence of a constellation of symptoms for a period ranging from 4 weeks from the onset of symptoms to 3 months (3). The symptoms presented in patients with this syndrome are myalgias, muscular dysfunction, sleep abnormalities, psychiatric and psychoemotional alterations, pulmonary fibrosis, and reduction in lung capacity (4, 6).

The evaluation of each patient with post-COVID-19 syndrome enrolled in some medical and physiotherapeutic treatment must include the state of consciousness, the function of the cardio-respiratory and musculoskeletal system (6). The purpose of this is to obtain a specific evaluation of the general condition after SARS-CoV-2 infection and design an appropriate plan of recovery (34). In this study, the patients were evaluated using a psycho-emotional test, a spirometry test, a 6-min talk test, and a hand dynamometer test in addition to a physical examination to explore the general condition before starting the treatment. Derived from the first evaluation we observed that the most frequent sequelae in our population were dyspnea (76%), fatigue (54%), and dry cough (28%), additionally, we found patients with severe (23%), moderate (22%), and mild (13%) anxiety as well as moderate (10%) and mild (22%) stress; these data are similar to those reported in other studies on COVID-19 patients (3, 35, 36).

The main point of this study was the evaluation of the physical therapy effects on patients with OSC ameliorating the physical sequelae and boosting their recovery. Here we proposed a physiotherapeutic treatment plan (Table 1) composed of 12 sessions to be employed in patients with the clinical stability to perform active mobilization (5). Even though the sequelae of this disease are heterogeneous, patients with COVID-19 develop several alterations in the respiratory mechanics, hence it is important to develop a general rehabilitation program focused on the muscular, respiratory, and cardiovascular systems, and based on the clinical necessities of each patient-derived from this disease. This protocol was developed following the next points as a basis: simple, safe, and functional. The objectives of the physiotherapeutic plan were: (1) retraining the breathing pattern, (2) improving respiratory and cardiovascular capacity, (3) increasing muscle strength, (4) reducing fatigue and dyspnea, (5) reintegrating the patient into daily activities using a gradual increase of aerobic, progressive strengthening (breath, core, and upper limb muscles), and breathing exercises as main tools (5, 11).

Results obtained after the medical reassessment of the patients after completing the 12 sessions of physiotherapy showed a reduction of at least 50% of the post-COVID sequelae, highlighting the benefits of this physiotherapy plan. These results could be attributed to the different therapeutic techniques performed in the physiological systems affected (3, 5). The DASS-21 test showed an improvement in the mental health status of the population enrolled in this study, obtaining an increase of 26% related to the mild score for anxiety, 44% in the mild stress score, and 18% in the mild depression score; however, the use of a single technique or test in the psycho-emotional evaluation must be interpreted with caution due to the reliability in the answers given by the patients (16).

In relation to what was found in the spirometry test, our results showed an improvement in FEV1 of 7.167%, in FVC of 7.561%, and a decrease in the FEV1/FVC ratio of −0.019. Zhu et al. reported an improvement in FVC at 4 weeks of 22.02%, 25.14% in FEV1, and 0.03 in the FEV1/FVC ratio (37). The decrease in the FEV1/FVC ratio that we obtained can be explained by the improvement in the FVC, it must be clinically relevant for the detection of patients with a risk factor such as an obstructive pathology since the FEV1/FVC ratio could be underestimated when the FVC is also affected by a restrictive pathology, this is the case in some patients with the post-COVID-19 syndrome. Esmaeilzadeh et al. described that 58.5% of patients “post-COVID-19” were affected by a syndrome they called “Asthma-like” defined by the presence of classic asthma symptoms, an FEV1/FVC ratio <80, improvement of 12%, and 200 ml in FEV1 after the administration of bronchodilator in which only 15.94% of the patients had symptoms prior to infection by the SARS-CoV2 virus (38). The abovementioned information is transcendental when the patient presents symptoms of obstruction, we should be less strict with the spirometry definition (FEV1/FVC <80) and give the patient the benefit of using an inhaled bronchodilator or even an inhaled steroid in the case of patients with “Asthma-like” even before rehabilitation. On the other hand, Lewis et al. in a multicenter study with patients who had spirometry before and after infection by SARS-CoV2 did not find a significant difference in the FVC (82% vs. 79.9%), FEV1 (73.1% vs. 73%), and FEV1/FVC (70.9% vs. 71.8%); this is probably explained by the fact that 48.8% of the patients had mild disease, 20% severe disease, and 0% critical disease. This agrees with the meta-analysis by Huan et al. where it was observed that the pre-and post-rehabilitation spirometric changes in patients with post-COVID-19 Syndrome were inconsistent in various studies (39, 40).

Derived from the performance and evaluation of the 6 MWT (maximal functional capacity, 6-min walk test performance, basal heart rate, resting oxygen saturation) in this study, it was possible to measure the effect of the treatment. In the maximal functional capacity test, we observed an improvement of 0.577 METs (One MET is defined as 1 kilocalorie per kilogram per hour and is the caloric consumption of a person while at complete rest), meaning an increase in the capacity to do their tasks (24, 41). Likewise, we observed an increase in the percentage of performance in the 6 MWT of 13%, and in the basal oxygen saturation, it was increased by 1.40% at the end of treatment. The basal heart rate increased by 2.36 beats per minute, being this result is not a significant change. Similarly, the results from the handgrip strength test showed an increase in both left hand and right hand of 2.90 and 2.24 Kg, respectively. These results can be taken together with the other data in this study to obtain a correct interpretation of the functionality of this protocol (42).

In conclusion, the physiotherapeutic plan and a medical multidisciplinary assessment in this work showed a decrease of about 50% of the post-COVID-19 symptoms-sequelae as well as an improvement in the psychoemotional status of the patients. From the spirometry test, we observed an increase of 7.16% in the FEV1 value and 7.56% for FVC. The maximal functional capacity improved by 0.577 METs, the 6-min walk test performance increased by 13%, and the basal saturation of oxygen-enhanced by 1.40%. Finally, the handgrip strength test showed an increase in both left hand and right hand of 2.90 and 2.24 Kg, respectively. We suggest that a physiotherapeutic plan should be implemented together with multidisciplinary medical support. Also, it is necessary an evaluation with different clinical tests to obtain the most accurate health status of each patient. We used this protocol following a physiotherapeutic methodology in the patients affected by COVID-19 in order to provide information on the treatment and management of the sequelae generated by the infection of SARS-CoV-2.

## Data Availability Statement

The raw data supporting the conclusions of this article will be made available by the authors, without undue reservation.

## Ethics Statement

The studies involving human participants were reviewed and approved by University Ethics Committee of Universidad Autónoma de Aguascalientes. The patients/participants provided their written informed consent to participate in this study.

## Author Contributions

SP-C and JD were the primary authors of the manuscript. DM-A planning and design of the physiotherapeutic protocol, 6-min walk test, and physical examination. AG-D was in charge of the area of physical therapy and execution of the physiotherapeutic protocol. PA-L and FA-G contributed to the planning and development of the project and FA-G was responsible for major funding of the project. FT-F and EH-C contributed to the data collection and revision of the manuscript. All authors contributed to the article and approved the submitted version.

## Funding

This work was supported by regular and special resources UAA for the Medical Didactic Unit, Health Sciences Center and Research 2021 and 2022.

## Conflict of Interest

The authors declare that the research was conducted in the absence of any commercial or financial relationships that could be construed as a potential conflict of interest.

## Publisher's Note

All claims expressed in this article are solely those of the authors and do not necessarily represent those of their affiliated organizations, or those of the publisher, the editors and the reviewers. Any product that may be evaluated in this article, or claim that may be made by its manufacturer, is not guaranteed or endorsed by the publisher.
